# 

**Published:** 1869-11

**Authors:** 


### OHIO STATE DENTAL SOCIETY.

The regular annual meeting of this body will be held at Columbus, on Tuesday, December 7th, at 10 o'clock, A. M., and continue in session probably two days.

We trust that this meeting will call together the entire available force of the profession of the State. There should be an attendance of two hundred, and there would be if there existed in the profession a proper interest for its progress and welfare. We have occasionally heard the excuse, "Oh I can't afford to go." The reply to such persons is, you can't afford to stay away, for if a man's professional ability is only able to just keep him from starving, the sooner he abandons the practice the better. Indeed, his neighbors can not afford to entrust such an one with the care of their teeth.

We regard it as beyond all question that he who will not avail himself of the advantages derivable from professional association and influence that goes out from it, will necessarily fail to attain a medium standing as a Dentist.

What a grand impetus would be given to the profession if every good man in the profession would come up to these meetings, prepared with heart and hand honestly to do his part of the work.

Let no one be afraid to bring up his secrets, let no one hesitate to show his ignorance; the best way many a time to get light is to let it be known that we are in darkness.

The profession in Ohio occupies a most enviable position, one in which there is encouragement to work, the results of which can not be otherwise than an abundance of fruit. Great things have been accomplished here, and still greater are expected. Shall these expectations be disappointed? Matters of great and vital importance will come before this meeting far consideration, and it is desirable that the whole profession in the State, so far as possible shall take part in the deliberation of such questions as may come up. We regard this as by far the most important meeting of our profession that has ever been held in the State, and we trust that no one that has the real good of his profession at heart, will, for any slight cause, suffer himself to be kept away. Let every active member be on hand, and every one who desires to be an active member, and every lobby member, and visitors, let them come. It is a duty they all owe to themselves, to theirprofession, present and prospective, and to their patients.

T.

---

To the Members of the American Dental Association.—Section 5 of Article III. of the new constitution of the American Association, adopted at its last session is as follows: "Permanent members shall consist of those who, having served one year as delegates, and complied with the requirements of the Association, and shall signify to the Treasurer, a desire for permanent, membership." I therefore request all members who have not already done so, to signify at once to me their desire, whether they wish to be considered permanent members, that I may adjust my books accordingly. Wm. H. Goddard, Pres.

Cor. 4th and Green Sts., Louisville, Ky.

